# Supplementary material for: Electrophysiology of hiPSC-Cardiomyocytes Co-Cultured with HEK Cells Expressing the Inward Rectifier Channel
Source: Int J Mol Sci. 2021 Jun 21;22(12):6621. doi: 10.3390/ijms22126621 (PMC8235371; doi:10.3390/ijms22126621)
Supplement: Supplementary file 1 [file ijms-22-06621-s001.zip › ijms-1224989-supplementary.pdf]

Figure S1

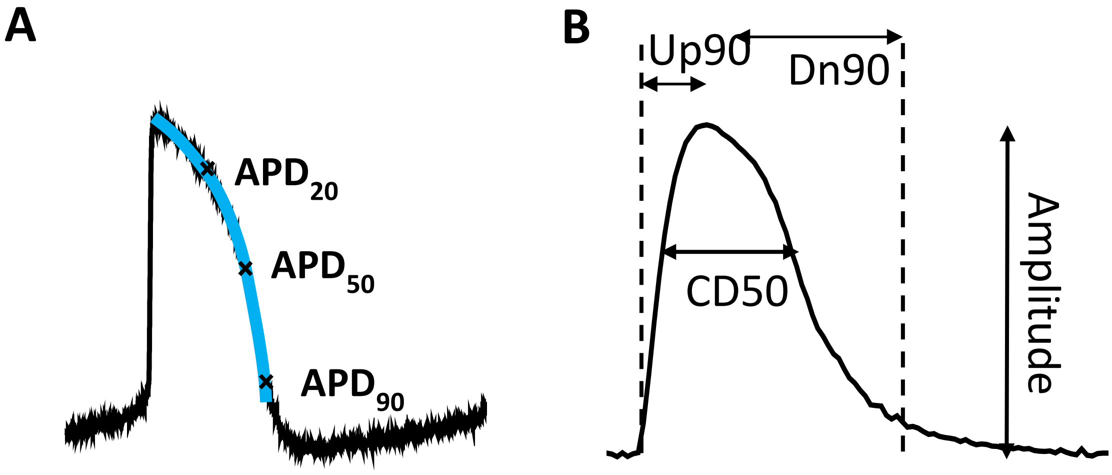

Figure S1 - Electrophysiology and contractility example traces. A) Example action potential trace displaying time to rise of AP (TRise) and different stages of the AP repolarisation: 20, 50 and 90% (APD20, 50, 90). B) Example of contractile trace showing the time for contraction (Up90), time for relaxation (Dn90), amplitude, and 50% of contraction duration (CD50).

Figure S2

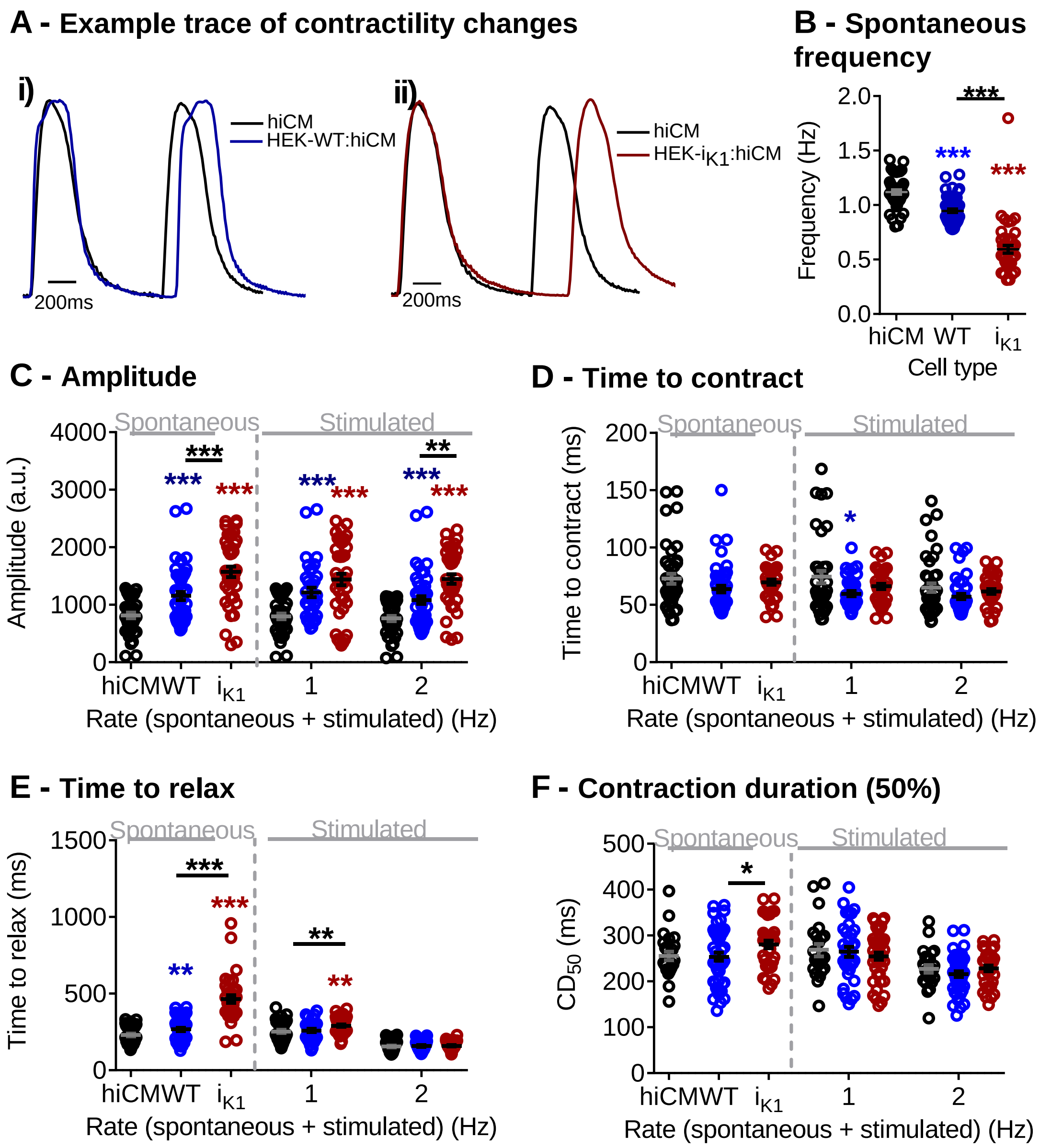

Figure S2 - Contractile behaviour is affected by the presence of HEK at 1:1 ratio with hiCM at day 4 in incubation recorded in serum-free solution. A) i) Example trace of contractility changes in the presence of HEK-WT:hiCM; ii) example trace of contractility in HEK-i_{K1}:hiCM ; B) Frequency of spontaneous contractions; C) time for contraction at spontaneous rates and electrically paced; D) time for relaxation; E) 50% contraction duration. One-way ANOVA vs. HEK-i_{K1} (red)/HEK-WT (blue). Unpaired t-test HEK-i_{K1} vs. HEK-WT (black): ***$p<0.001$, **$p<0.01$, *$p<0.05$, N=5 plates, n=48 wells.

Figure S3

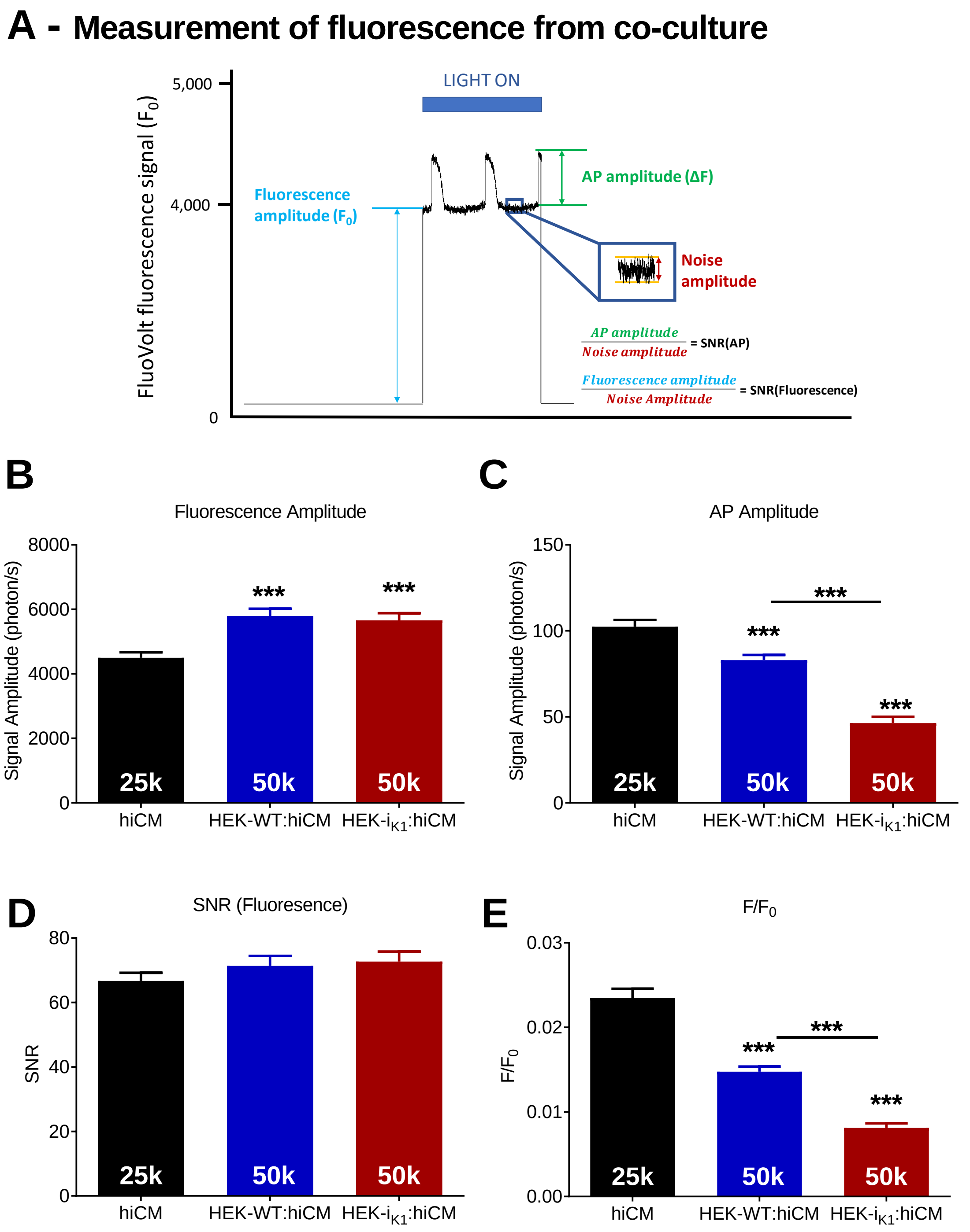

Figure S3 - Signal-to-noise calculated by measuring the fluorescence amplitude collected from 470nm excitation using a voltage-sensitive dye and/or the AP amplitude for HEK:hiCM at 1:1. A) There is little or no fluorescence on CellOPTIQ while the excitation light is off, and this increases immediately when the light is on (blue). The fluorescence amplitude can be measured as a baseline for the AP trace (green), and AP amplitude corresponds to the size of the AP minus the fluorescence amplitude (orange). The noise amplitude is measured from the bottom to the highest point of the noise between each AP (yellow). B) Amplitude of the baseline fluorescence; C) The amplitude of the signal/AP measured from baseline to peak of AP; D) SNR of the fluorescence; E) SNR of the AP. White shows total number of cells in culture. Unpaired t-test comparing co-culture to hiPSC-CMs, and between different co-cultures; ***p<0.001, **p<0.01, n=32 wells (Corrected for Poisson noise).

Figure S4

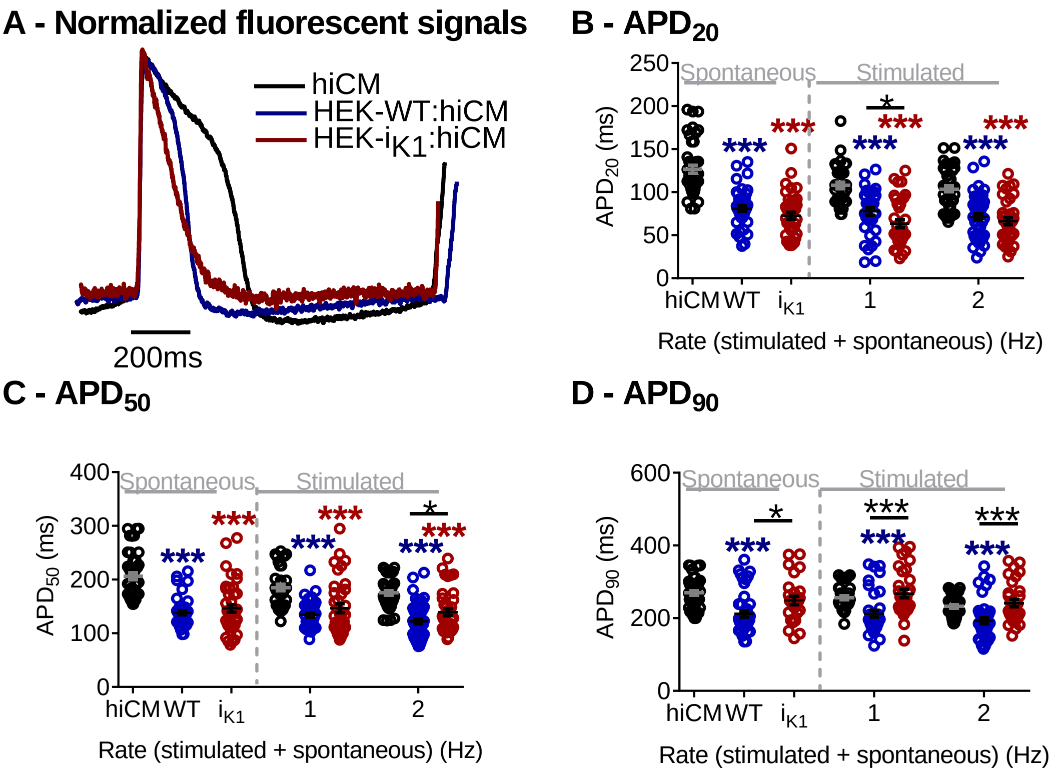

Figure S4 - Electrophysiological behaviour is affected by the presence of HEK at 1:1 HEK:hiCM. A) Example trace of normalized APs in the presence of HEK-WT and in HEK- i_{K1} at spontaneous rates; B) effects on early repolarisation (20%) at spontaneous rate and electrical stimulation; C) mid-repolarisation (50%); D) late repolarisation at 90%. One-way ANOVA CMs vs. HEK-i_{K1} (red)/HEK-WT (blue). Unpaired t-test HEK-i_{K1} vs. HEK-WT (black). ***<0.001, **p<0.01, *p<0.05, N=5 platings, n=48 wells. (Amplitude analysis is shown separately (Figure S3).

Table S1

Table S1 - The effects of co-culture on spontaneous electrical activity. Spontaneous AP rates were affected by the presence of HEK. At lower stimulation rates (1Hz) percentage of cultures not following is affected by the intrinsic spontaneous rate, which is similar for hiCMs and HEK-WT:hiCM. Higher percentages of cultures not following at higher pacing rates related to the cells becoming inexcitable in the presence of i_{K1}.

Culture	Spontaneous rates (Hz)	Spontaneous (quiescence)	1Hz (% wells not following)	2Hz (% wells not following)	3Hz (% wells not following)
hiCM	1.20±0.05	0%	13%	0%	3%
1:1 HEK-WT:hiCM	0.91±0.01	0%	19%	0%	6%
1:1 HEK-iK1:hiCM	0.68±0.03	19%	25%	19%	47%

Figure S5

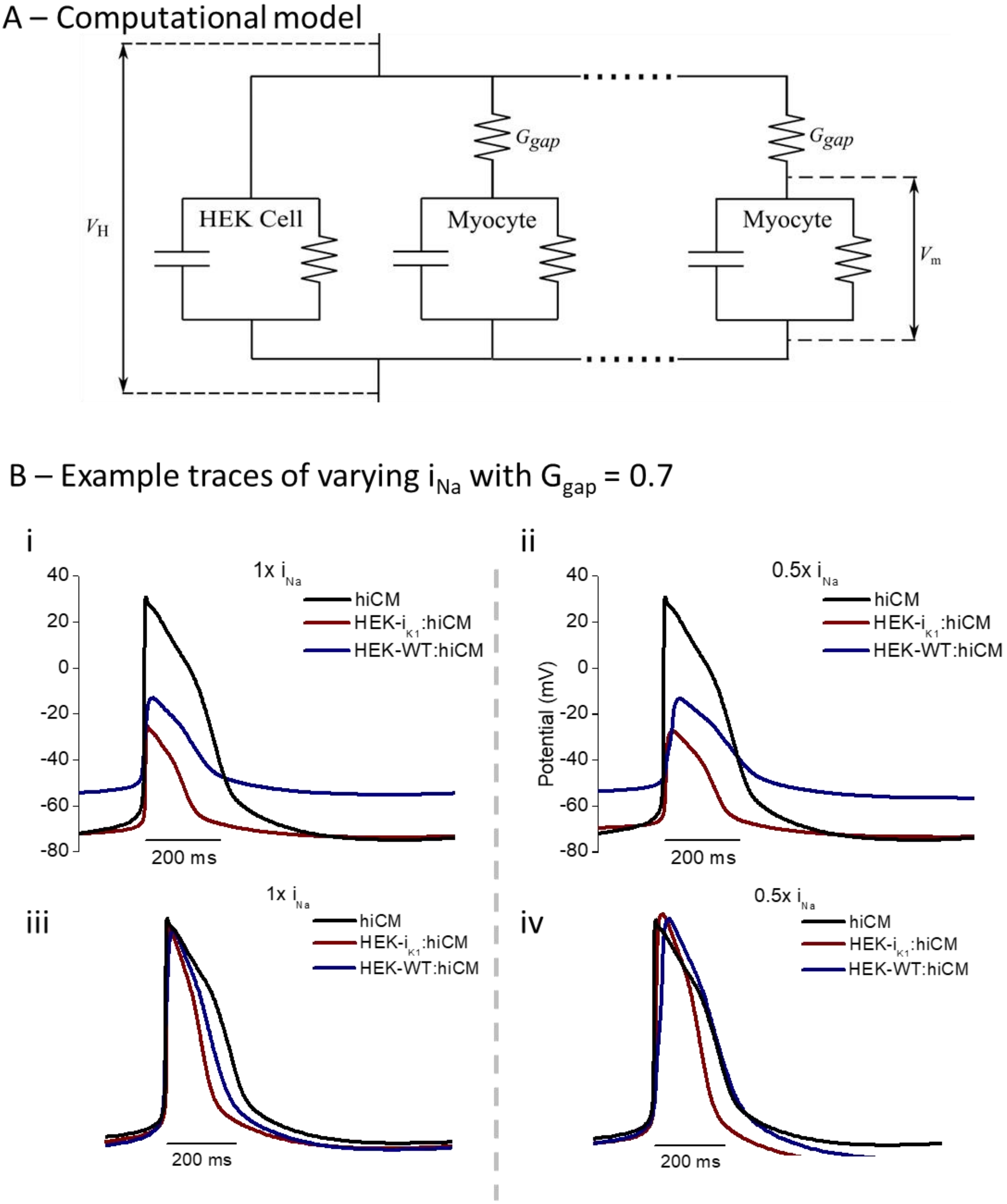

Figure S5 – Computational model was created based on the circuit presented in A). Example APs when Ggap =0.7nS are presented in B): i) APs for hiCM and co-culture of hiCM with either HEK-WT or HEK-i_{K1} using a full i_{Na} model; ii) APs for hiCM and co-culture of hiCM with either HEK-WT or HEK-i_{K1} using a half i_{Na} model; iii) normalised APs in full i_{Na} show the effects of co-culture on electrophysiology; iv) normalised APs in half i_{Na} show the effects of co-culture on electrophysiology.

Figure S6

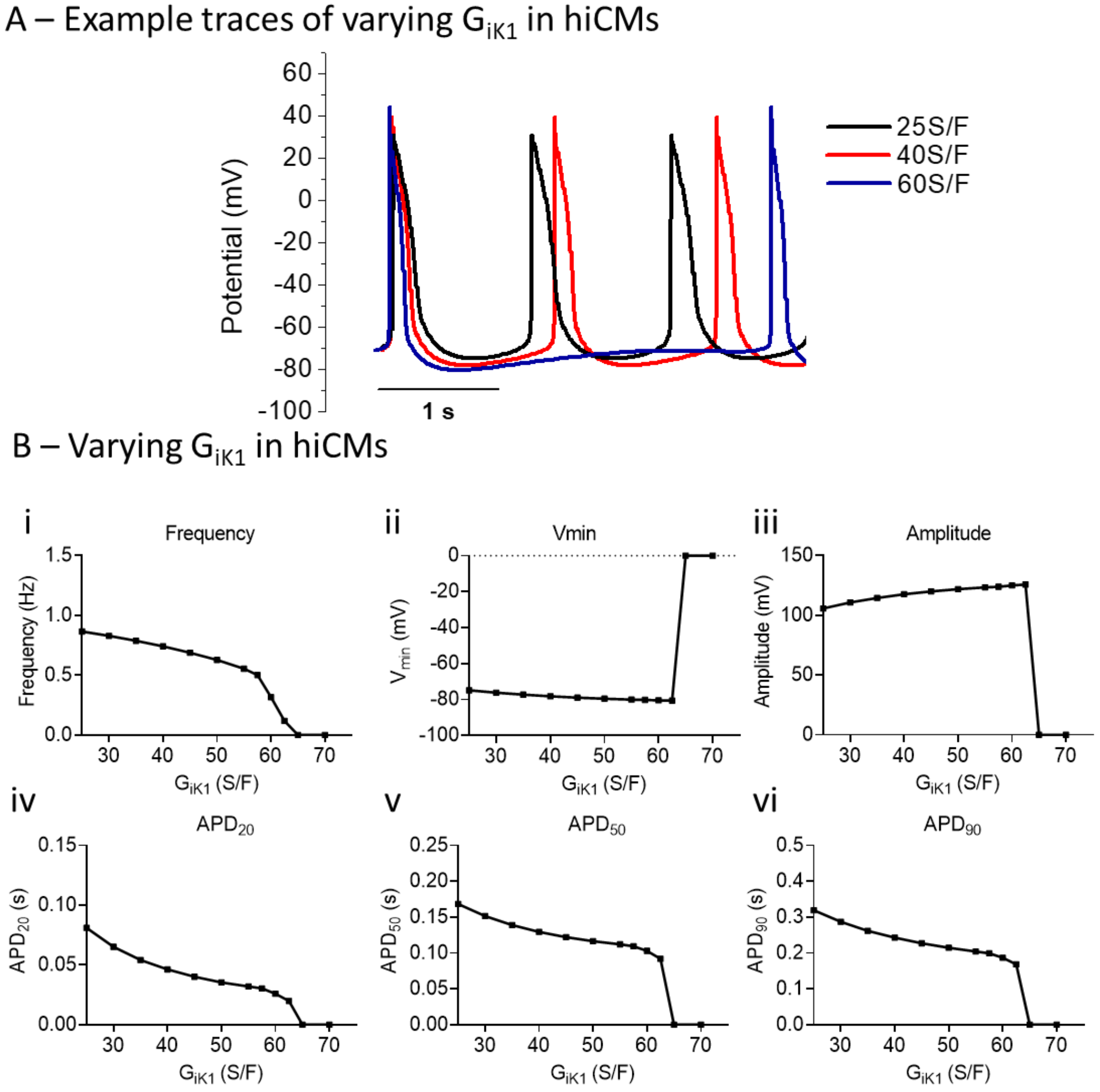

Figure S6 – Varying iK1 conductance in CM using computational modelling. A) Example traces of the effects of varying GiK1 at 25S/F, 40S/F and 60S/F on potential; B) i) effects of increasing GiK1 on frequency of spontaneous contraction; ii) effects on diastolic potential (Vmin); iii) varying amplitude; iv, v and vi) effects on early, mid and late repolarisation.

Figure S7

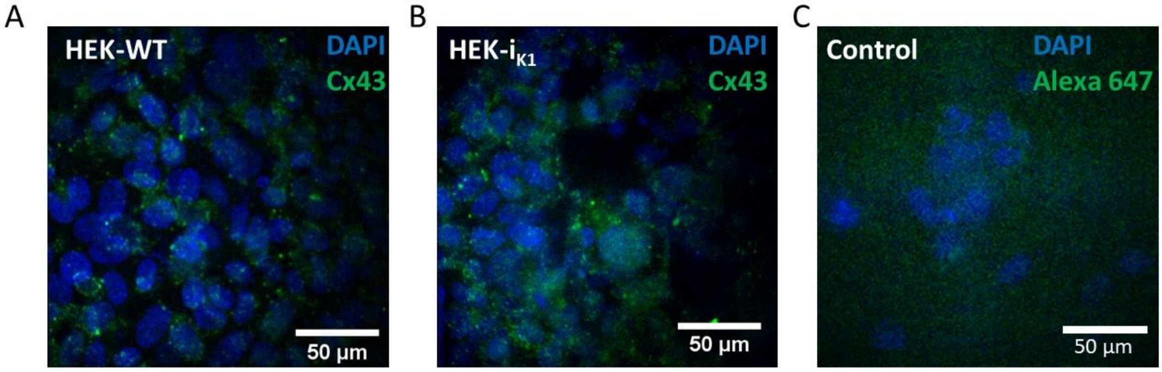

Figure S7 – Presence of Connexin43 in HEK-WT and HEK-I_{K1} cells. A) HEK-WT. B) HEK-I_{K1}. C) Control (no Cx 43 antibody). Cx43 staining (Green) and DAPI (Blue)

Methods:

HEK to be used for immunocytochemistry were plated in 6-well glass-bottom plates (MatTek) and incubated for a minimum of 8 days at 37C, 5% CO2. Existing medium was removed from the cells, and the culture was washed 3 times with PBS (-Ca2+ -Mg2+). The cells were fixed for 15 minutes at RT with 4% PFA and washed 3 times with 0.1% PBST (PBS + 0.1% Tween20). The cells were then permeabilised with 0.1% Triton X-100 diluted in PBS for 10 minutes at RT, and washed 3 times in PBST for 5 minutes each wash. The culture was then blocked for 60 minutes with 10% goat serum in PBS. The block was removed, and the cells were incubated overnight with 1:200 anti-connexin 43 antibody (rabbit) (Merck, Germany) in PBST + 10% goat serum. The cells were further washed 3 times in PBST at RT with 5 minutes for each wash and incubated for 1h with anti-rabbit Ig-Alexa 647 at 1:200 dilution in PBST + 10% goat serum. The supernatant was removed and PBST with 1:1000 DAPI was added for 3 minutes. The cells were then washed 3 times with PBST before imaging. A control HEK culture was stained in the same manner, but without the anti-connexin 43 antibody. Stained cells were placed on an Olympus IX83 confocal microscope under 40x (N.A. 0.6, air objective) magnification. Illumination was switched to 637 nm laser light (Obis continuous wave laser, Coherent, USA) with a 700/75 nm emission filter for visualisation of Cx43. 405 nm laser light (Obis continuous wave laser, Coherent, USA) was used for visualisation of the nucleus (DAPI). Minimal laser power was used in order to prevent significant photobleaching and a standard exposure time of 200 ms and an EM gain of 300 was used for the majority of images but was adjusted if required. Images were processed using ImageJ (NIH) to subtract background and merge channels.
